# Supplementary material for: A New Insight into the Bactericidal Mechanism of 405 nm Blue Light-Emitting-Diode against Dairy Sourced Cronobacter sakazakii
Source: Foods. 2021 Aug 25;10(9):1996. doi: 10.3390/foods10091996 (PMC8470084; doi:10.3390/foods10091996)
Supplement: Supplementary file 1 [file foods-10-01996-s001.zip › foods-1339188-supplementary.pdf]

Supplementary Materials:

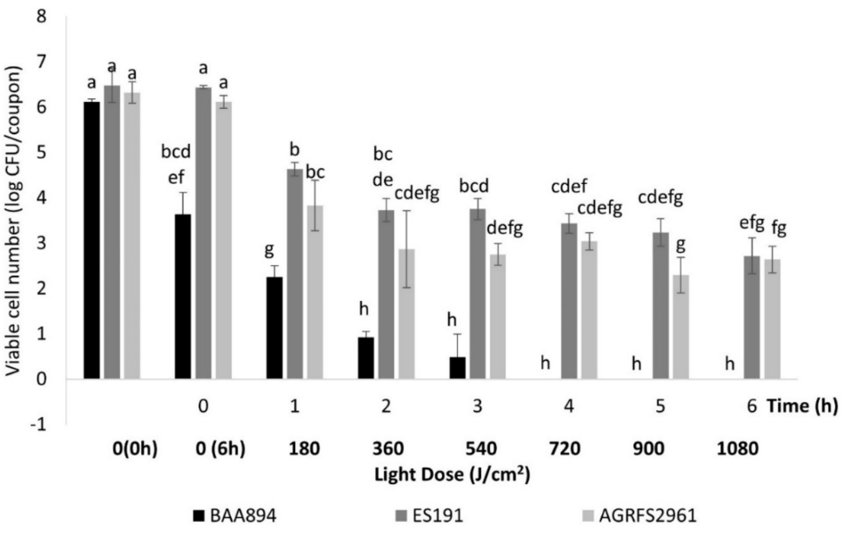

Figure S1. The antimicrobial effect of 1080J/cm2 light exposure against three-day-biofilm cells (grown from 10% skim milk) on stainless steel coupon. Groups that share the same letters are insignificantly different. The 3-day-old biofilm on each coupon was grown from 10% skim milk (mentioned in method 2.7.2). Biofilms were washed in 30mL of PBS three times to remove curdled milk and unattached bacterial cells. Eight biofilm-containing coupons were used in the experiment for each strain. One coupon was swabbed immediately for estimating the number of biofilm cells at the beginning (0 h). Six coupons were illuminated for different times (1–6 h) before sampling for examining the number of viable biofilm cells. The last coupon was the control group without light illumination for 6 h. The sampling of biofilm cells is mentioned in method 2.7.2 and the experiment was done in triplicate.

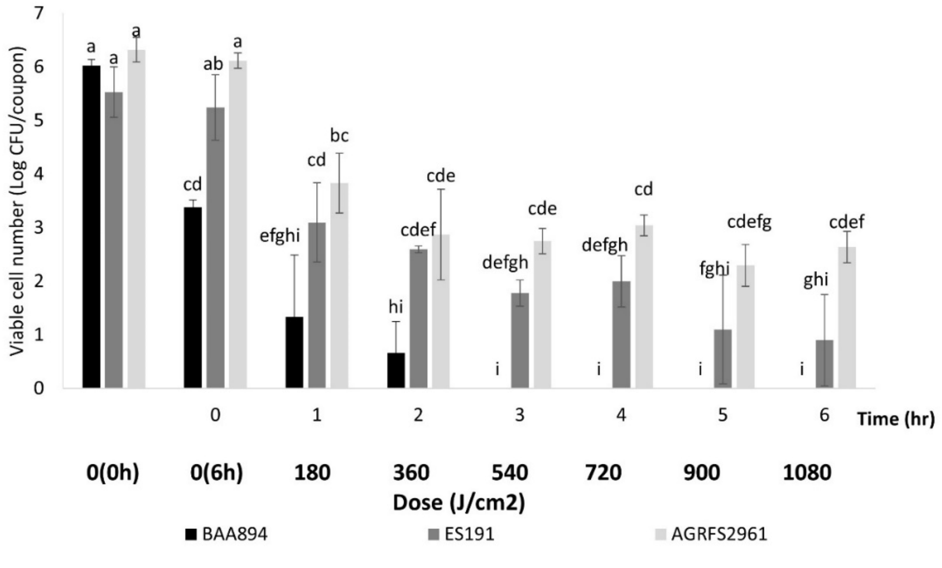

Figure S2. The antimicrobial effect of 1080J/cm2 light exposure against three-day-biofilm cells (grown from 10% skim milk) on POM plastic coupon. Groups that share the same letters are insignificantly different. The biofilms on plastic

coupon were obtained using the same method mentioned in Supplementary Figure 1.

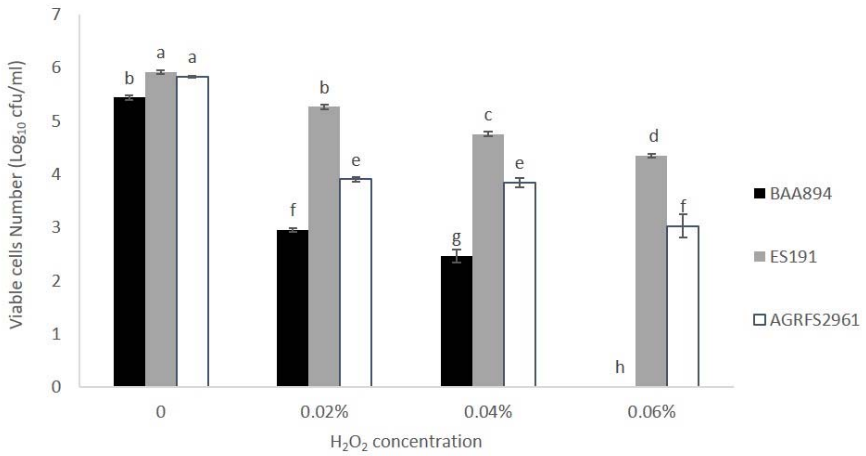

Figure S3. Cell survival of three tested *C. sakazakii* strains when facing with different concentration of H_2O_2 for a 4-h incubation at 25°C. Groups that share the same letters are insignificantly different. Overnight culture cells from 10% skim milk were resuspended and diluted in PBS. One ml aliquots of 6 log CFU/mL cell suspension containing 0, 0.02%, 0.04%, and 0.06% H_2O_2 (final concentrations) were added into each well of a 24 well plate and incubated for 4 h at 25 °C. Cell suspensions containing no H_2O_2 were used as control and incubated under the same condition in the test. After the experiment, cell suspension in each well was sampled and washed immediately for examining the number of viable cells, was enumerated by plate counting (serial dilution for plating on TSB agar for 48 h incubation at 37 °C).

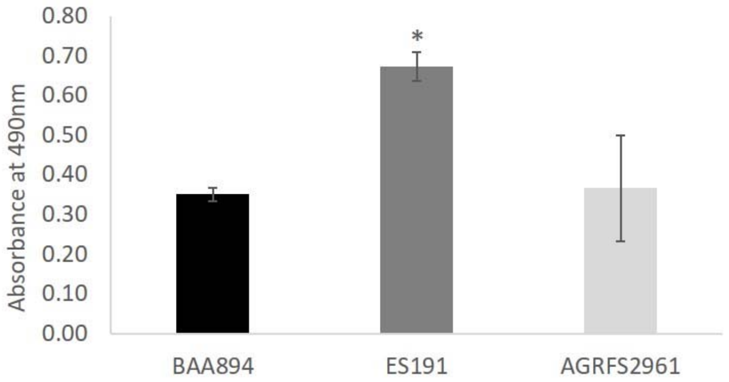

Figure S4. The comparison of extracellular polysaccharides substance (EPS) mass. Congo red was used to evaluate EPS reduction in a published study [15]. For each strain, three-day-old biofilms on the well bottom of six well plates (Nunclon Delta™) were washed with PBS, and 1ml of congo red (50μM in RO water) was added into each well. The congo solution incubated with biofilms was pipetted into new tubes and centrifuged at 7000 × g for 2 min. The supernatants (200 μL) were transferred into a new 96 well plate and colorimetric change was measured by spectrophotometry reader at 490nm (Varioskan™ LUX, Thermo Scientific).
